# Draft genome sequence of 16 *Aspergillus flavus* isolated from cashew nuts from coastal Kenya

**DOI:** 10.1128/mra.00445-24

**Published:** 2024-09-09

**Authors:** Pauline Wambui Gachanja, Manase Aloo Onyango, Colletah Rhoda Musangi, Bicko Steve Juma, Dennis Wamalabe Mukhebi, Eugene Mwanza Muzami, Kyalo Katua, Wilton Mwema Mbinda

**Affiliations:** 1 Department of Biochemistry and Biotechnology, Pwani University, Kilifi, Kenya; 2 Pwani University Bioscience Research Centre PUBReC, Pwani University, Kilifi, Kenya; University of Strathclyde, Glasgow, United Kingdom

**Keywords:** *Aspergillus flavus*, cashew nut, draft genome, aflatoxigenic

## Abstract

*Aspergillus flavus* is a soil-borne fungus known for its aflatoxin contamination of agricultural products. Here, we report the draft genome sequences of 16 predicted aflatoxin-producing *A. flavus* isolated from cashew nuts from coastal Kenya.

## INTRODUCTION

*Aspergillus flavus* is a filamentous saprophytic fungus that contaminates important agricultural products, including cashew nuts (1). Under favorable conditions, such as drought and heat stress, *A. flavus* produces secondary metabolites, such as aflatoxins (2). Aflatoxins are carcinogenic, hepatotoxic, and can cause aspergillosis in immunocompromised individuals (2, 3). Aflatoxin contamination in crops poses significant threats to global food safety, particularly in sub-Saharan Africa, which has warm and humid climates that are conducive to aflatoxin production (4, 5). Aflatoxin contamination of crops is expected to increase due to climate change shocks (6). Here, we present draft genome sequences of 16 predicted aflatoxigenic *A. flavus* isolated from cashew nuts (Table 1), which is essential in understanding *A. flavus* phylogenetics and comparative and functional genomics.

**TABLE 1:** Genome sequences of *Aspergillus flavus* sp. isolated from cashew nuts from coastal Kenya

Parameter/Sample No.	10B	11B	12A	13B	15A	16A	17A	18B	19B	1B	20B	22B	4B	5B	7B	9A
Genbank accession No.	JBAWIZ000000000	JBAWIU000000000	JBAWIT000000000	JBAWIS000000000	JBAWIR000000000	JBAWIQ000000000	JBAWIP000000000	JBAWIO000000000	JBAWIN000000000	JBAWIK000000000	JBAWIM000000000	JBAWIL000000000	JBAWIX000000000	JBAWIY000000000	JBAWIV000000000	JBAWIW000000000
SRA accession No.	SRR28841496	SRR28841501	SRR28841488	SRR28841489	SRR28841490	SRR28841491	SRR28841492	SRR28841493	SRR28841494	SRR28841503	SRR28841495	SRR28841502	SRR28841498	SRR28841497	SRR28841500	SRR28841499
Number of reads	21,050,726	22,806,790	17,032,196	20,990,232	19,001,446	17,128,796	21,850,708	19,905,174	20,603,930	19,419,956	20,553,698	21,310,018	#########	20,196,650	22,126,146	19,341,246
Genome size (bp)	37,449,923	37,283,523	37,243,444	37,657,964	37,316,005	37,274,043	39,804,705	37,649,694	38,097,618	37,177,891	38,422,971	37,308,414	37,559,519	37,759,462	37,310,799	37,307,524
Genome coverage (×)	83.4	90.8	67.9	82.7	75.5	68.2	78.1	78.4	80.2	79.3	84.7	77.5	92.4	79.4	88	76.9
% G + C	47.5	47.5	47.5	47.5	47.5	47.5	47.5	47.5	47.5	48	47.5	47.5	47.5	47.5	47.5	47.5
No. of contigs	63	87	158	164	78	126	11,715	183	609	437	859	80	102	127	66	81
N50 contigs	1.4 Mb	1.2 Mb	993 kb	894.4 kb	1.1 Mb	988.7 kb	58.1 kb	894.4 kb	775 kb	1 Mb	384.5 kb	899.9 kb	903.4 kb	978.4 kb	931.3 kb	1.1 Mb
No. of scaffolds	25	58	122	122	38	80	9,715	135	560	399	760	36	65	84	29	48
N50 scaffolds	2.5 Mb	1.9 Mb	1.8 Mb	1.7 Mb	1.9 Mb	2.1 Mb	271.9 kb	2.1 Mb	983.3 kb	1.8 Mb	579.5 kb	2 Mb	1.6 Mb	2.1 Mb	2.1 Mb	2.4 Mb
Total no. of Busco orthologs	1,706	1,706	1,706	1,706	1,706	1,706	1,706	1,706	1,706	1,706	1,706	1,706	1,706	1,706	1,706	1,706
Complete single-copy, complete multicopy, fragmented, and missing orthologs (%)	98.6, 0.4, 0.0, 1.0	98.5, 0.4, 0.0, 1.0	98.5,0.4, 0.0, 1.1	98.6, 0.4, 0.0, 1.0	98.5,0.4, 0.0, 1.1	98.7, 0.4, 0.0, 0.9	93.1, 0.5, 3.2, 3.2	98.5,0.4, 0.0, 1.1	98.6, 0.4, 0.0, 1.0	98.5, 0.5, 0.0, 1.0	98.4, 0.5, 0.1, 1.0	98.7, 0.4, 0.0, 0.9	98.7, 0.4, 0.0, 0.9	98.5, 0.5, 0.0, 1.0	98.6, 0.4, 0.0, 1.0	98.6, 0.4, 0.0, 1.0
ITS BLAST similarity (%), reference match and accession	*Aspergillus flavus, 95,* MT573498.1.	*Aspergillus flavus, 91,* MT071404. 1	*Aspergillus niger, 98,* MK372989.1	*Aspergillus flavus, 99,* OK086056. 1	*Aspergillus flavus, 95,* MK992255.1		*Aspergillus flavus, 85,* MT994594.1	*Aspergillus flavus, 94,* MK992254. 1	*Aspergillus sp.,* OK210353. 1	*Aspergillus flavus, 99,* KX067886. 1	*Aspergillus terreus, 98,* KJ685810.1				*Aspergillus aculeatus, 99,* KP965728.1	
28s rRNA BLAST similarity (%), reference match and accession	*Aspergillus novoparasitic us, 99,* NG069972. 1	*Aspergillus oryzae, 99,* KX958066. 1	*Aspergillus sp., 99,* MN515285. 1	*Aspergillus flavus, 98,* MT509808. 1	*Aspergillus flavus, 99,* MT252035. 1	*Aspergillus flavus, 95,* MT509808. 1	*Aspergillus flavus, 99,* MT252035. 1	*Aspergillus novoparasitic us, 99,* NG069972. 1		*Aspergillus flavus, 100,* MT509808. 1	*spergillus terreus, 99,* MH877949. 1	*Aspergillus aculeatus, 99,* MK518351. 1	*Aspergillus novoparasitic us, 99,* NG066672. 1	*Aspergillus novoparasiticus, 100,* MT252035.1	*Aspergillus aculeatus, 99,* MH870630. 1	*Aspergillus aculeatus, 99,* JQ301899.1
Calmodulin BLAST similarity (%), reference match and accession	*Aspergillus flavus, 99.48,* KY272751.1	*Aspergillus flavus, 98.81,* LS999591.1	*Aspergillus flavus, 98.48,* MK304471.1	*Aspergillus flavus, 99.48,* MN271387.1	*Aspergillus flavus, 99.14,* LC061194.1	*Aspergillus flavus, 99.66,* MG991523.1	*Aspergillus flavus,* 99.48, KY272751.1	*Aspergillus flavus,* 99.14, MG991523.1	*Aspergillus flavus,* 99.65, MN271387.1	*Aspergillus flavus,* 91.40, MN271387.1	*Aspergillus flavus,* 98.95, LC061194.1	*Aspergillus flavus,* 98.62, LS999591.1	*Aspergillus flavus,* 99.65, MN271387.1	*Aspergillus flavus,* 98.97, HF570041.1	*Aspergillus flavus,* 98.80, MG991523.1	*Aspergillus flavus,* 99.37, MN416023.1

Cashew nut samples were collected in coastal Kenya’s production areas (Kilifi, Kwale, and Lamu) in May 2021 (7, 8). Whole cashew nuts were surface sterilized with 70% ethanol. Cashew shells were cut into four sections, and the kernels were cut into approximately 3 × 4-mm pieces. The pieces were directly plated on modified Rose Bengal Agar medium and incubated at 30°C for 7 days in darkness. Fungi growing were transferred to Water Agar medium and incubated for 3 days at 27°C in the light. The resulting hyphae were cultured on potato dextrose agar (PDA) and malt extract agar media at 25°C for 7 days under light to isolate pure cultures. Species identification was based on morphological determination, Sanger sequencing of PCR products of ITS (ITS1/ITS4), and 28s rRNA regions (NL1/NL4) (9), and calmodulin gene (Cmd5/Cmd6) (S1) (10) at Macrogen, Netherlands. Pure isolates were further cultured on PDA at 25°C for 7 days under darkness. Resulting mycelia were used for DNA extraction using ZR Fungal/Bacterial DNA Miniprep kit (Zymo, Irvine, USA). The sequences were queried using NCBI BLASTn v2.14.0 (11) (Table 1).

**Fig 1:**
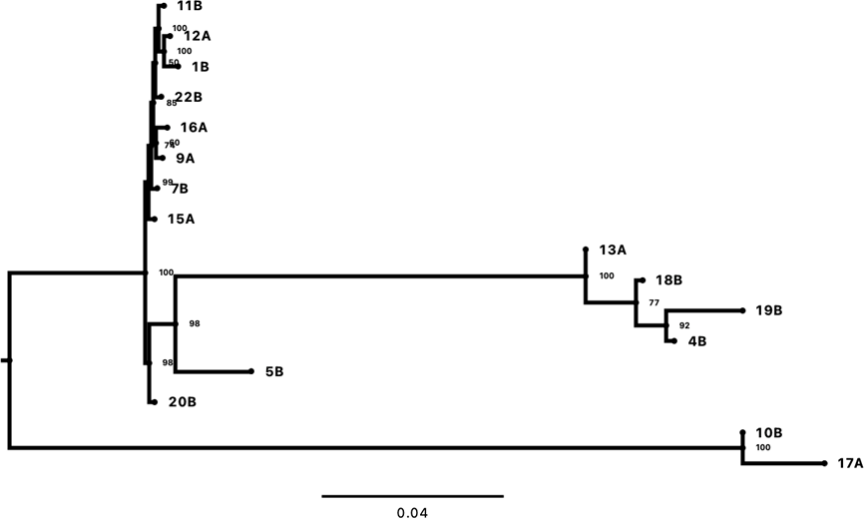
Phylogenetic tree of 16 predicted aflatoxin-producing *Aspergillus flavus de novo* genomes isolated from cashew nuts from coastal Kenya. The tree was constructed using the maximum likelihood algorithm in IQ-TREE v2.2.2.7 with the TVM + F + I + G4 model.

Sequencing library of the 16 *A*. *flavus* predicted to produce aflatoxin (12) was generated by TruSeq DNA PCR-Free kit (Illumina, San Diego, USA), and short reads paired-end genome sequencing was carried out using Illumina’s NovaSeq-6000 at Macrogen, South Korea. FASTQC v0.12.1 was used to check the quality of the paired-end (PE) raw reads (13). PE raw reads were filtered using fastp v3.3.5 to eliminate reads with Q scores of <20 and adapters (14). The reads had an average length of 151 bp, and the total number of reads for each genome are listed in (Table 1). Clean reads were used for *de novo* genome assembly using SPAdes v3.15.4 with the “careful” option and k-mer sizes 21, 33, 55, 77, and 99 (15). SPAdes contigs and scaffolds are shown in Table 1. Completeness of the genome assemblies (Table 1) was determined using BUSCO v5.7.1 with the lineage database ascomycota_odb10 (16). The diversity of the *A. flavus* genomes was illustrated with phylogenetic analysis using maximum likelihood algorithm in IQTREE v2.2.2.7 with TVM + F + I + G4 model (17). The phylogenetic tree and midpoint rooting (Fig. 1) was generated using Figtree v1.4.4 (18). Default parameters were used for all software unless otherwise specified.

## Data Availability

The whole genome sequences of the 16 *A. flavus* were deposited in the NCBI Genbank project under Bioproject number PRJNA1051575. The GenBank and Sequence Read Archive (SRA) accession numbers are listed in Table 1. This is the first announcement of *A. flavus* draft genome sequences from cashew nuts from coastal Kenya.
